# Three-year outcomes of endovascular repair of descending thoracic aortic lesions with a single-branched endograft for left subclavian artery preservation

**DOI:** 10.1016/j.xjse.2025.100096

**Published:** 2025-12-19

**Authors:** G. Chad Hughes, Michael D. Dake, Himanshu J. Patel, Jon S. Matsumura, Ali Azizzadeh, Nimesh Desai, William T. Brinkman, Dennis Gable, Sukgu Han, Gustavo Oderich

**Affiliations:** a Department of Surgery, Duke University Medical Center, Durham, NC; b Department of Medical Imaging, University of Arizona Health System, Tucson, Ariz; c Department of Cardiac Surgery, University of Michigan Hospitals, Ann Arbor, Mich; d Department of Surgery, University of Colorado School of Medicine, Aurora, Colo; e Division of Vascular Surgery, Cedars-Sinai Medical Center, Los Angeles, Calif; f Division of Cardiovascular Surgery, Department of Surgery, University of Pennsylvania, Philadelphia, Pa; g Baylor Scott and White, The Heart Hospital, Plano, Tex; h Division of Vascular Surgery and Endovascular Therapy, Keck Medical Center of University of Southern California, Los Angeles, Calif; i Division of Vascular Surgery and Endovascular Therapy, Baylor College of Medicine, Houston, Tex

**Keywords:** aortic arch, left subclavian artery, endovascular, branch endograft, outcomes

## Abstract

**Objective:**

Zone 2 thoracic endovascular aortic repair with a single-branched endograft (GORE TAG Thoracic Branch Endoprosthesis [TBE], W.L. Gore & Associates, Inc) was approved by the Food and Drug Administration in May 2022 on the basis of 1-year results from a pivotal trial. This report presents 3-year trial outcomes in patients with nondissection descending thoracic aortic lesions requiring zone 2 proximal landing zone.

**Methods:**

In this prospective, nonrandomized cohort study across 40 US sites, 106 adults underwent thoracic endovascular aortic repair using the TBE device, which includes a side-branch for left subclavian artery perfusion. Three cohorts were evaluated: aneurysm (AN; n = 84), traumatic aortic injury (TAI; n = 9), and other isolated lesions (OL; n = 13).

**Results:**

Mean ages were AN 70 ± 11, TAI 42 ± 19, and OL 65 ± 13 years; male patients comprised 63%, 89%, and 46%, respectively. At 36 months, all-cause mortality was 14% overall (AN 13%, TAI 0%, OL 31%) with 1 lesion-related death (1%). Late (>30 days) stroke occurred in 5%. Core laboratory−adjudicated imaging follow-up revealed 8% type I/III endoleaks and 99% left subclavian artery branch patency. Aortic enlargement (>5 mm) was observed in 5% (all in AN cohort; none requiring reintervention), with no instances of aortic rupture, device migration, or wire fracture reported. Late reinterventions were required in 7% of patients, all in the AN group.

**Conclusions:**

At 3 years, the TBE device demonstrated durable outcomes for nondissection descending thoracic aortic disease, with high branch patency, low lesion-related mortality, and minimal complications across diverse pathology cohorts. These results support its role as a safe, effective alternative to surgical revascularization in anatomically suitable zone 2 cases.

## Graphical Abstract

**Figure:**
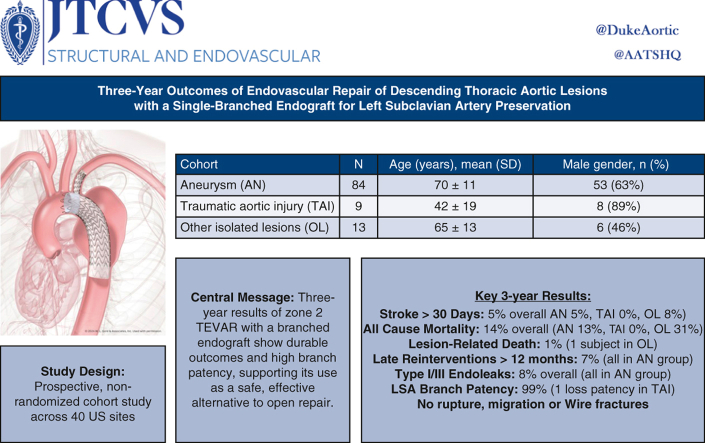

**Figure:**
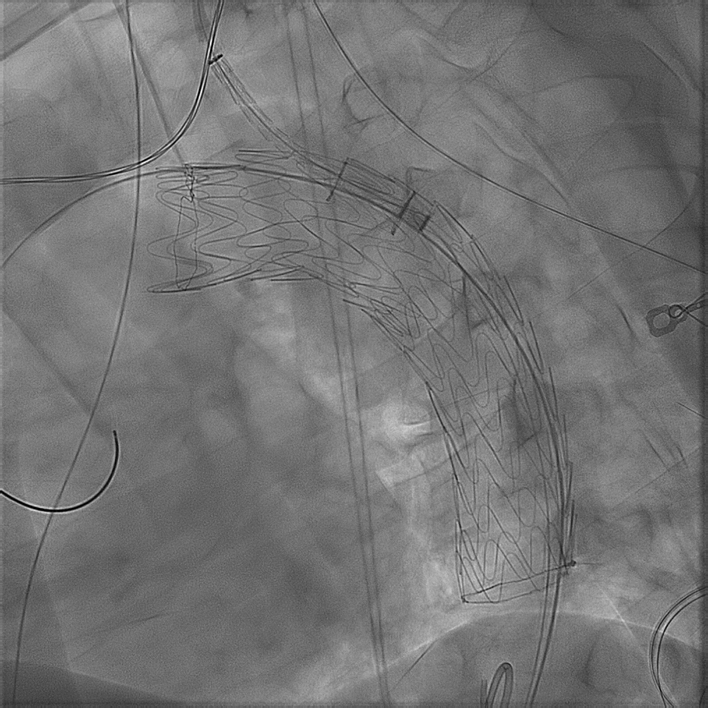
Fluoroscopic image of deployed TBE device with LSA branch in place prior to deployment.

**Central Message**

Three-year results of zone 2 TEVAR with a branched endograft show durable outcomes and high branch patency, supporting its use as a safe, effective alternative to open repair.

**Perspective**

This report presents the first 3-year outcomes of zone 2 TEVAR using a single-branched endograft for left subclavian artery preservation in nondissection lesions. Results demonstrate durable performance with high branch patency, low lesion-related mortality, and minimal complications, supporting its role as a safe alternative to open surgery.

The GORE TAG Thoracic Branch Endoprosthesis (TBE; W.L. Gore and Associates) is the only commercially available device approved by the Food and Drug Administration in the United States for zone 2 thoracic endovascular aortic repair (TEVAR) with endovascular preservation of left subclavian artery (LSA) flow via a side branch. The device was approved in May 2022 and consists of an aortic endograft with an internal portal for a side branch consisting of a nitinol stent covered with expanded polytetrafluoroethylene fabric.^1^, ^2^, ^3^ Device approval was based on the results of a nonrandomized, prospective pivotal trial, and 1-year outcomes for the TBE device in treating aneurysms,^1^ dissections,^2^ and blunt traumatic aortic injury^3^ have been previously reported. The purpose of the current report is to present midterm (3-year) results from the TBE pivotal trial cohort, specifically for nondissection lesions of the descending thoracic aorta requiring zone 2 proximal seal zone, including aneurysm, traumatic aortic injury (transection),^3^ and other isolated lesions (intramural hematoma, penetrating aortic ulcer, Kommerell diverticulum, or pseudoaneurysms which met criteria for TBE treatment but did not fit within the other cohorts).^4^

## Methods

**Figure 1:**
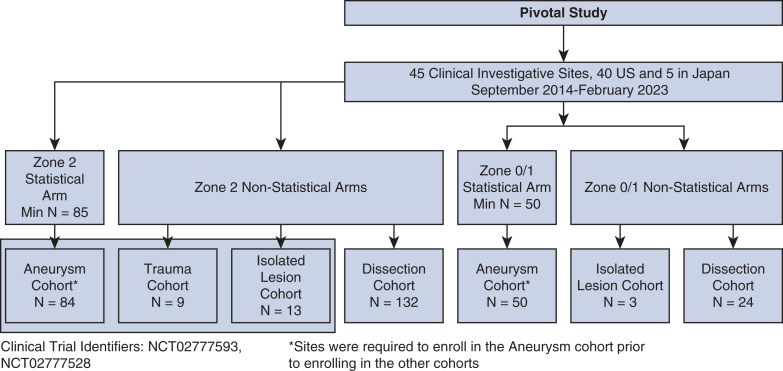
Consolidated Standards of Reporting Trials diagram of the Thoracic Branch Endoprosthesis pivotal study. The nondissection zone 2 cohorts presented in the current report are denoted by a *black box* at the bottom left of the diagram.

Across 45 investigative sites, including 40 in the United States and 5 in Japan, this prospective, nonrandomized, multicenter study of patients with lesions of the descending thoracic aorta requiring placement of a proximal endovascular stent-graft with retrograde oriented portal and single side branch was designed with 4 independent arms: zone 2 and zone 0/1 aneurysm cohorts and zone 2 and zone 0/1 nonaneurysm (traumatic aortic injury, dissection, or other lesion types) cohorts, for a total of 7 cohorts (no trauma cohort in zone 0/1). A Consolidated Standards of Reporting Trials diagram of the trial is presented in Figure 1. For the current report of patients with zone 2 nondissection only, 40 enrolling sites, all in the United States, participated with a data lock of April 10, 2024.

A total of 559 patients in the overall zone 2 arm were screened, and 250 were enrolled. On the basis of the defined eligibility criteria, the most common reasons for screen failure were inadequate proximal aortic (48%) or target branch vessel (13%) landing zones. After the exclusion of the screen failures, there were 84 enrolled patients included in the zone 2 aneurysm (AN) cohort (n = 43 fusiform, n = 41 saccular), 9 in the traumatic aortic injury (TAI) cohort, and 13 in the other isolated lesions (OL) cohort, yielding a total study population of 106 adult patients with nondissection descending thoracic aortic lesions requiring zone 2 proximal landing zone. The zone 2 dissection cohort (n = 136) have not yet completed 3 years of follow-up and will be detailed in a future report.

The trial protocol and procedures were approved at each participating institution by their individual institutional review boards, and each site obtained informed consent for enrolled patients. Enrollment in the zone 2 cohort of the trial occurred between September 2016 and October 2019. The study protocol is available online at ClinicalTrials.gov (Clinical Trial Identifier: NCT02777593) and uploaded in the Online Data Supplement.

**Figure 2:**
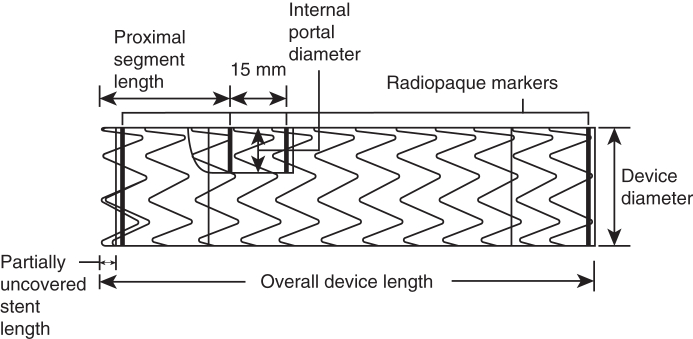
Diagram of the Thoracic Branch Endoprosthesis aortic component. The proximal segment length refers to the distance from the leading edge of the internal portal to the leading edge of the partially uncovered stents on the proximal end of the device. For the 8-mm internal portal device, this length varies from 20-25 mm, whereas for the 12-mm internal portal device the length is 40 mm.

**Figure 3:**
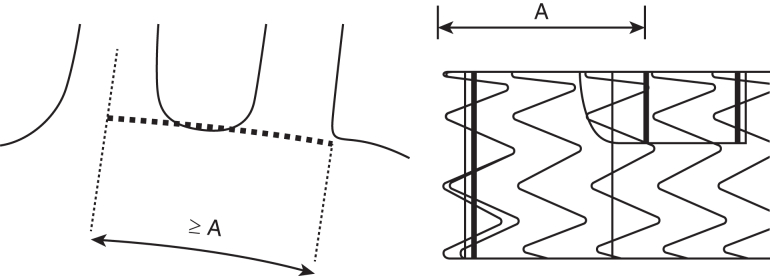
Proper positioning of the proximal end of the TBE device. The partially uncovered portion of the aortic component, the length of which varies from 3 to 6.5 mm depending on device diameter, may be positioned to cover up to one half the diameter of the left common carotid artery ostium or bovine origin with zone 2 deployment. The *dotted line* in the *left image* represents the proximal seal zone in zone 2 being measured along the outer curve of the aorta between the distal edge of the left subclavian artery to the midpoint of the left common carotid artery, which is unique to the TBE device. *TBE*, Thoracic Branch Endoprosthesis.

Device characteristics and details of the implantation procedure have been previously described.^1^, ^2^, ^3^ The main aortic component device comes in diameters ranging from 21 to 45 mm and will treat intended aortic diameters from 16 to 42 mm. Furthermore, the device is available in 2 configurations, one with an 8-mm internal portal and a proximal segment length of 20 to 25 mm and the other with a 12-mm internal portal and a proximal segment length of 40 mm (Figure 2). Optional aortic extenders are likewise available in the same diameters as the main aortic component but are shorter and range in length from 3.6 to 4.6 cm, as they are intended for short-segment proximal seal zone extension. Assessment of covered length of the proximal seal zone in zone 2, measured obtained along the outer curve of the aorta between the distal edge of the LSA to the midpoint of the left common carotid artery, is unique to the TBE device (Figure 3).

Study end points were as previously described,^1^, ^2^, ^3^ and the 12-month primary end point data for the aneurysm^1^ and nonaneurysm^2^^,^^3^ zone 2 cohorts have been previously reported. The current report details midterm (36-month) outcomes for patients enrolled in the AN, TAI, and OL cohorts. Per the study protocol, patient follow-up evaluations, including contrast-enhanced computed tomographic angiography, were scheduled at 1, 6, and 12 months and then annually through 5 years posttreatment as previously described.^1^, ^2^, ^3^

For analysis of imaging-based outcomes, visit windows were defined per protocol as follows: procedure (0 days); postprocedure (1-14 days); 1 month (15-59 days); 6 months (60-242 days); 12 months (243-546 days); 24 months (547-911 days); and 36 months (912-1275 days). For outcomes not requiring imaging, windows were defined as follows: 30 days (0-30 days); 6 months (31-182 days); 1 year (183-365 days); 2 years (366-731 days); and 3 years (732-1096 days). Thus, the 36-month window extends through 1275 days.

A multidisciplinary data and safety monitoring board periodically reviewed the study data for patient safety, and an independent clinical events committee adjudicated the study end points. An independent core laboratory reviewed the follow-up computed tomographic angiography imaging data from 1-month through end of follow-up at 60 months. Endoleaks were classified according to prespecified trial definitions, which are detailed in Table E1.

Because of the lack of an open surgical control group in the trial, this represents an observational and largely descriptive report, and no formal comparisons were conducted as the result of limited statistical power. Summary data are presented as proportions for categorical variables and as mean ± standard deviation for continuous variables. Results were generated using SAS, Version 9.4, System for Windows (SAS Institute).

## Results

Patient demographics and comorbidities are presented in Table 1. Mean patient age was 70 ± 11 years in the AN cohort, 42 ± 19 years in TAI, and 65 ± 13 in OL; male patients comprised 63%, 89%, and 46% of the 3 cohorts, respectively. Patient comorbidities in the AN and OL cohorts included multiple comorbidities typical of the distal aortic surgery population, whereas the younger TAI cohort had fewer comorbidities as expected. Thirty-eight percent of patients in the AN cohort and 54% of the OL cohort had undergone previous aortic surgery, including surgery involving the proximal aorta (AN cohort: 12% previous ascending, 6% arch; OL cohort: 23% ascending, 15% arch), descending thoracic aorta (AN cohort only: 2%), or abdominal aorta (AN cohort: 17%; OL cohort: 23%). Forty-four percent of patients (51% AN cohort; 0% TAI cohort; 31% OL cohort) required distal TEVAR extension at the time of the index procedure, in addition to the TBE device, to completely exclude the entirety of the aortic pathology within the descending thoracic aorta.

**Table 1:** Patient demographics and comorbidities

	Aneurysm n = 84	Trauma n = 9	Other isolated lesions n = 13
Age, y, mean (SD)	70 (11)	42 (19)	65 (13)
Male sex, n (%)	53 (63%)	8 (89%)	6 (46%)
BMI, kg/m^2^, mean (SD)	28.8 (6.3)	29.5 (5.0)	25.8 (5.3)
Comorbidities, n (%)			
Hypertension	72 (86%)	4 (44%)	11 (85%)
Diabetes mellitus	14 (17%)	1 (11%)	2 (15%)
Hypercholesterolemia	44 (52%)	1 (11%)	6 (46%)
Coronary artery disease	27 (33%)	1 (11%)	3 (23%)
Peripheral vascular disease	11 (13%)	1 (11%)	1 (8%)
Previous stroke	12 (14%)	1 (11%)	1 (8%)
Nicotine use	30 (36%)	2 (22%)	7 (54%)
Chronic obstructive pulmonary disease	16 (19%)	0 (0%)	3 (23%)
Previous aortic repair (any segment)	32 (38%)	0 (0%)	7 (54%)

*BMI*, Body mass index; *SD*, standard deviation.

For the overall cohort of 106 patients, 16 died during follow-up and 20 were lost to follow-up by 36 months. This left 78 (73.5%) patients eligible for evaluation within the 3-year window, of whom 63 (80.7%) completed a follow-up visit and 59 (75.6%) underwent computed tomography imaging. Midterm device outcomes, as assessed by 3-year complete core laboratory−adjudicated imaging follow-up, included a single (1%; n = 1/104) loss of LSA branch patency at 6 months, which did not require revascularization, and 4 (5%; n = 4/84) cases of late aortic enlargement >5 mm, none of which required reintervention. There were no instances of late aortic rupture, device migration, or wire fracture (Table 2). Thirty-six percent (n = 37/104) of patients had an endoleak noted during follow-up, most commonly type II (21%, n = 22/104) or indeterminate (18%, n = 19/104), with type I (3%, n = 3/104) or III (5%, n = 5/104) endoleaks occurring much less commonly (Table 3). No type Ic endoleaks related to the side branch were observed. Core laboratory adjudication indicated that the type II endoleaks originated from intercostal or bronchial arteries. Clinical events committee−adjudicated late reinterventions occurred in 7% (n = 7/106) of patients, all in the AN cohort, including redo-TEVAR for endoleak (n = 2: 1 type IIIa and 1 type Ia; at postoperative days [PODs] 420 and 840 after index TBE), redo-TEVAR for new distal type B dissection (n = 2, PODs 303 and 1179) or intramural hematoma (n = 1, POD 405), LSA revascularization (n = 1, POD 1197 for LSA stenosis with patent LSA branch), and ascending aorta and hemiarch replacement for acute type A dissection (n = 1, POD 64).

**Table 2:** Core laboratory−reported device events through 3 years∗

	Aneurysm n = 84†‡	Trauma n = 9†‡	Other isolated lesions n = 13‡	Total nondissection cohorts n = 106†‡
SB loss of patency	0/82 (0%)	1/9 (11%)	0/13 (0%)	1/104 (1%)
Aortic rupture	0/83 (0%)	0/9 (0%)	0/13 (0%)	0/105 (0%)
Device migration	0/84 (0%)	0/9 (0%)	0/13 (0%)	0/106 (0%)
Wire fracture	0/84 (0%)	0/8 (0%)	0/13 (0%)	0/105 (0%)
Aortic enlargement (>5 mm)§	4/66 (6%)	0/8 (0%)	0/10 (0%)	4/84 (5%)

∗ Table data include event findings related to the TBE as well as any distally implanted Conformable GORE TAG Thoracic Endoprosthesis (CTAG) thoracic devices through 1275 days postoperatively.

† There were 2 subjects excluded from this analysis (1 with a zone 2 aneurysm and 1 with a zone 2 traumatic aortic injury) because having a clinical events committee−adjudicated major inclusion/exclusion criteria violation or Food and Drug Administration−mandated exclusion.

‡ For core laboratory assessments, study window through 3 years is 1 to 1275 days. Denominators for each device finding category are the number of evaluable subjects (had either computed tomography angiography or magnetic resonance angiography imaging) with a known evaluation in window.

§ Aortic enlargement is based on maximum transverse diameter of aneurysm/lesion. Subjects evaluated for change from baseline are those subjects that have both a baseline measurement and a measurement in each follow-up time window. If more than 1 nonmissing measurement in a time window, the largest (worst) aneurysm diameter used for analysis.

**Table 3:** Core laboratory−adjudicated endoleaks and reinterventions in zone 2 nondissection cohorts through 3 years∗

	Aneurysm n = 84	Trauma n = 9	Other isolated lesions n = 13	Total nondissection cohorts n = 106†
Any endoleak‡	36/82 (44%)	0/9 (0%)	1/13 (8%)	37/104 (36%)
Type I	3/82 (4%)	−	0/13 (0%)	3/104 (3%)
Type II	21/82 (26%)	−	1/13 (8%)	22/104 (21%)
Type III	5/82 (6%)	−	0/13 (0%)	5/104 (5%)
Indeterminate	19/82 (23%)	−	0/13 (0%)	19/104 (18%)
CEC-adjudicated reinterventions	7/84 (8%)	0/9 (0%)	0/13 (0%)	7/106 (7%)

∗ Table data include core laboratory−adjudicated endoleaks and clinical events committee−adjudicated reinterventions through 1275 days postoperatively.

† There were 2 subjects excluded from this analysis (1 with a zone 2 aneurysm and 1 with a zone 2 traumatic aortic injury) due to having a CEC-adjudicated major inclusion/exclusion criteria violation or Food and Drug Administration−mandated exclusion.

‡ For core laboratory assessments, study window through 3 years is 1 to 1275 days. Denominators for each device finding category are the number of evaluable subjects (had either computed tomography angiography or magnetic resonance angiography imaging) with a known evaluation in window.

All-cause mortality in the 106 patients through 3 years was 14% (n = 15), with only 1 (1%) aortic-related death (lesion-related mortality) (Table 4). This occurred in a patient in the OL group at 534 days after the index TBE procedure, which had been performed for intramural hematoma. Death was attributable to a de novo type B aortic dissection in the setting of a new distal stent graft-induced aortic wall injury,^5^ which resulted in aortic rupture and death while subject was awaiting reintervention as previously described.^4^ The 14 late nonaortic deaths included cardiac-related (n = 5; POD range, 97-901), cerebrovascular-related (n = 2; POD 129 and 166), respiratory failure (n = 2; POD 167 and 875), infection (n = 3; POD range, 79-1066), cancer (n = 1; POD 368), and failure to thrive (n = 1; POD 641). There were a total of 5 late strokes beyond 30 days, including 4 in the AN cohort (n = 4/84, 5%) and 1 in the OL cohort (n = 1/13, 8%).

**Table 4:** Late mortality and strokes in zone 2 nondissection cohorts through 3 years∗

	Aneurysm n = 84†	Trauma n = 9†	Other isolated lesions n = 13†	Total nondissection cohorts n = 106†
All-cause mortality	11/84 (13%)	0/9 (0%)	4/13 (31%)	15/106 (14%)
Lesion-related mortality	0/84 (0%)	0/9 (0%)	1/13 (8%)	1/106 (1%)
Late (>30 d) stroke	4/84 (5%)	0/9 (0%)	1/13 (8%)	5/106 (5%)

∗ Study window through 3 years is 0-1096 days.

† There were 2 subjects excluded from this analysis (1 with a zone 2 aneurysm and 1 with a zone 2 traumatic aortic injury) due to having a clinical events committee−adjudicated major inclusion/exclusion criteria violation or Food and Drug Administration−mandated exclusion.

## Discussion

This report provides the first comprehensive 3-year follow-up on the use of the GORE TAG TBE for zone 2 thoracic endovascular aortic repair in patients with nondissection descending thoracic aortic lesions. The results demonstrate that the TBE device continues to perform reliably in the midterm, with excellent preservation of branch vessel patency, low lesion-related mortality, and minimal device-related complications.

One of the most important findings of this study is the durability of the LSA branch. At 36 months, LSA branch patency was maintained in 99% of patients, with only 1 case of late occlusion. The single loss of LSA branch patency was an asymptomatic occlusion noted in a patient in the TAI group at 6 months postoperatively and which did not require reintervention. This was believed to be a technical issue, as previously detailed,^3^ but does highlight concerns about the long-term patency of branch stents in the typically young trauma population. Reassuring, however, is the fact that the branch patency rate observed in the current study is similar to the 97% 5-year primary patency rate reported by Voigt and colleagues^6^ for patients undergoing carotid-subclavian bypass and the 100% patency at 2.8 years reported by Bianco and colleagues^7^ for carotid-subclavian transposition, both in the setting of zone 2 TEVAR. This longer-term success in preserving LSA perfusion supports the TBE device as a viable and less-invasive alternative to surgical revascularization,^8^ particularly in anatomically suitable patients. Previous publications^1^, ^2^, ^3^ have focused on 1-year outcomes, and the current data provide important midterm validation of this technology.

Lesion-related mortality was low (1%) across all cohorts, with no observed cases of late aortic rupture. Despite the presence of endoleaks in more than one third of patients, the majority of which were type II or indeterminate, aortic-related events remained rare. The 18% incidence of core laboratory−adjudicated indeterminate endoleaks largely reflected technical limitations of site-provided imaging, particularly suboptimal slice thickness that precluded definitive source identification, rather than a true biologic phenomenon. Consistent with this, none of the type II or indeterminate endoleaks resulted in aortic sac enlargement requiring reintervention, which aligns with previous reports in the literature^9^^,^^10^ and current consensus guidelines,^11^ which do not recommend intervention in the absence of significant (≥10 mm) sac growth. Of note, the presence of the LSA branch does introduce the potential for type IIIc endoleak, an issue that cannot occur with conventional nonbranched TEVAR, and 3 of the 5 type III endoleaks observed were type IIIc endoleaks involving the LSA branch stent as adjudicated by the core laboratory, although none required reintervention. Because postdeployment ballooning of the side branch portal is mandated in the TBE device Instructions for Use, and all implanting physicians were required to follow the Instructions for Use during this premarket trial, ballooning technique was not considered the source of these leaks. Rather, the classification as type III by the core laboratory was based on proximity to the side-branch portal in the absence of definitive imaging evidence linking the leak to the side branch itself or to the proximal seal zone.

The low reintervention rate (7%) underscores the durability of the TBE construct, with no instances of device migration, fracture, or structural failure reported. Notably, all of the reinterventions occurred in the aneurysm cohort, and more than one half of these (n = 4/7) were attributable to progression of native aortic disease rather than device-related complications. This progression was observed exclusively in the older, more comorbid AN and OL cohorts, emphasizing the importance of continued post-TEVAR surveillance, even in the absence of proximal device issues,^4^ particularly in patients with degenerative aortic pathology. In contrast, patients treated for traumatic aortic injury demonstrated excellent outcomes with no mortality and minimal complications through 3 years, including no instances of distal endograft infolding or collapse as have been previously reported with the use of earlier generation TEVAR devices in TAI.^12^ These findings support the use of the TBE device as a first-line option in this younger, low-comorbidity population and suggest that follow-up protocols could be individualized, with less-frequent imaging potentially appropriate given their younger age and greater susceptibility to cumulative radiation exposure.^13^^,^^14^

Finally, late stroke occurred in 5% of patients, all in the older and more comorbid aneurysm and other lesion cohorts. Although these events were not temporally related to the TBE procedure, they underscore the persistent cerebrovascular risk in this population. Most previous studies have focused on neurologic outcomes within the first 30 days^1^, ^2^, ^3^; in contrast, our extended follow-up highlights the continued cardiovascular risks in these patients. To address this, all follow-up visits should include not only imaging, but also aggressive medical management such as titration of antihypertensive therapy and ensuring statin adherence to mitigate long-term risk.^14^

In summary, this report presents comprehensive 3-year follow-up data on the use of the GORE TAG TBE for zone 2 TEVAR in patients with nondissection descending thoracic aortic lesions. The findings demonstrate durable midterm performance, characterized by excellent branch vessel patency, low lesion-related mortality, and minimal device-related complications. Although future studies with larger cohorts and longer follow-up are needed to validate these outcomes and assess durability beyond 3 years, the current data offer strong evidence supporting the midterm safety and efficacy of the TBE device in this nondissection patient population.

### Webcast

You can watch a Webcast of this AATS meeting presentation by going to: https://www.aats.org/resources/three-year-outcomes-of-endovas-10310.

**Figure:**
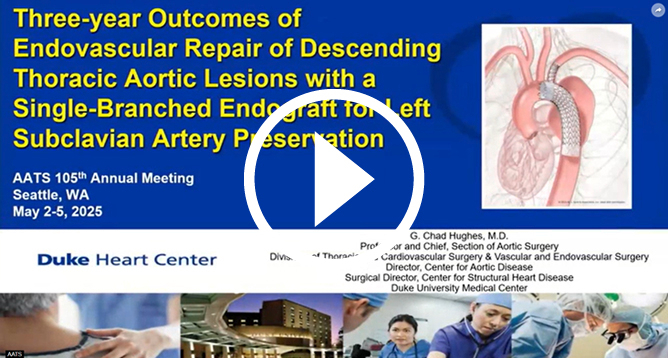

### Audio

You can listen to the discussion audio of this article by going to the supplementary material section below.

## Conflict of Interest Statement

G.C.H. reported consultant, speaker, and scientific advisory board: W. L. Gore & Associates, Inc. M.D.D. reported consultant: REVA Medical, scientific advisory board: W. L. Gore & Associates, Inc. J.S.M. reported research support: W. L. Gore & Associates, Inc. A.A. reported scientific advisory board (honorariums paid directly to institution): W. L. Gore & Associates, Inc. N.D. reported consultant: W. L. Gore & Associates, Inc, Artivion, Terumo Aortic, and Edwards Life Sciences. W.T.B. reported consultant, research support: W. L. Gore & Associates, Inc, Artivion, and Terumo Aortic. D.G. reported consultant, speaker, scientific advisory board: W. L. Gore & Associates, Inc, research support, speaker: Medtronic. S.H. reported consultant, scientific advisory board: W. L. Gore & Associates, Inc, Cook Medical, Terumo Aortic, consultant: Medtronic, scientific advisory board: Vestek and Taurus. G. O reported consultant, scientific advisory board: W. L. Gore & Associates, Inc, Cook Medical, Centerline, GE Healthcare, Shapeform Medical, and VITAA. All other authors reported no conflicts of interest.

The *Journal* policy requires editors and reviewers to disclose conflicts of interest and to decline handling or reviewing manuscripts for which they may have a conflict of interest. The editors and reviewers of this article have no conflicts of interest.

## Figures and Tables

**Table E1:** Endoleak type definition from GORE TAG Thoracic Branch Endoprosthesis (TBE) US Pivotal Trial

Type	Endoleak location
I	Type I endoleak is defined as a sealing failure at one of the attachment sites of the graft to the vessel wall Type Ia: proximal leak Type Ib: distal leak Type Ic: inadequate seal at distal landing zone for side branch component Arterial flow therefore leaks alongside the graft and into the perigraft space.
II	Type II endoleak is defined as retrograde flow from branch arteries arising from the treated segment of the aorta.
III	Type IIIa endoleak is referred to flow from module disconnection. Type IIIb endoleak is flow from fabric disruption.
IV	Type IV endoleak is defined as flow through porous fabric.
Indeterminate	Endoleak detected with unknown source.

## Supplementary Data

### Audio

Audio 1 This is an audio recording of the presentation of the associated abstract and discussion. The abstract presentation ends at 7:19 and the discussion begins at 7:25.

Video 1 Video available at: https://www.jtcvs.org/article/S2950-6050(25)00055-5/fulltext.

## References

[1] Dake M.D., Brinkman W.T., Han S.M. (2022). Outcomes of endovascular repair of aortic aneurysms with the GORE thoracic branch endoprosthesis for left subclavian artery preservation. J Vasc Surg.

[2] Desai N.D., Wang G.J., Brinkman W.T. (2025). Outcomes of a novel single branched aortic stent graft for treatment of type B aortic dissection. Ann Thorac Surg.

[3] Chou E.L., Lu E., Dake M.D. (2024). Initial outcomes of the gore TAG thoracic branch endoprosthesis for endovascular repair of blunt thoracic aortic injury. Ann Vasc Surg.

[4] Hughes G.C., Dake M.D., Patel H.J. (2025). Two-year outcomes of endovascular repair of isolated thoracic aortic lesions using a single-branch thoracic endograft with left subclavian artery preservation. J Thorac Cardiovasc Surg Open.

[5] Doberne J.W., Sabe A.A., Vekstein A.M. (2022). Stent graft-induced aortic wall injury: incidence, risk factors, and outcomes. Ann Thorac Surg.

[6] Voigt S.L., Bishawi M., Ranney D. (2019). Outcomes of carotid-subclavian bypass performed in the setting of thoracic endovascular aortic repair. J Vasc Surg.

[7] Bianco V., Sultan I., Kilic A. (2020). Concomitant left subclavian artery revascularization with carotid-subclavian transposition during zone 2 thoracic endovascular aortic repair. J Thorac Cardiovasc Surg.

[8] Squiers J.J., DiMaio J.M., Schaffer J.M. (2022). Surgical debranching versus branched endografting in zone 2 thoracic endovascular aortic repair. J Vasc Surg.

[9] Ranney D.N., Cox M.L., Yerokun B.A. (2018). Long-term results of endovascular repair for descending thoracic aortic aneurysms. J Vasc Surg.

[10] Squizzato F., Antonello M., Modena M. (2024). Fate of primary and indeterminate target vessel endoleaks after fenestrated-branched endovascular aortic repair. J Vasc Surg.

[11] Czerny M., Grabenwöger M., Berger T. (2024). EACTS/STS guidelines for diagnosing and treating acute and chronic syndromes of the aortic organ. Ann Thorac Surg.

[12] Canaud L., Alric P., Marzelle J. (2010). Factors favoring stent-graft collapse after thoracic endovascular aortic repair. J Thorac Cardiovasc Surg.

[13] White H.A., Macdonald S. (2010). Estimating risk associated with radiation exposure during follow-up after endovascular aortic repair (EVAR). J Cardiovasc Surg (Torino).

[14] Iribarne A., Keenan J., Benrashid E. (2017). Imaging surveillance after proximal aortic operations: is it necessary?. Ann Thorac Surg.

